# Neurite Outgrowth-Promoting Compounds from the Petals of *Paeonia lactiflora* in PC12 Cells

**DOI:** 10.3390/molecules27227670

**Published:** 2022-11-08

**Authors:** Takeru Koga, Hideyuki Ito, Yuji Iwaoka, Toshiro Noshita, Akihiro Tai

**Affiliations:** 1 Graduate School of Advanced Technology and Science, Tokushima University, 2-1 Minamijosanjima-cho, Tokushima 770-8506, Japan; 2 Faculty of Health and Welfare Science, Okayama Prefectural University, 111 Kuboki, Soja, Okayama 719-1197, Japan; 3 Faculty of Life and Environmental Sciences, Prefectural University of Hiroshima, 5562 Nanatsuka-cho, Shobara, Hiroshima 727-0023, Japan; 4 Department of Pharmacy, Gifu University of Medical Science, 4-3-3 Nijigaoka, Kani, Gifu 509-0293, Japan; 5 Graduate School of Technology, Industrial and Social Sciences, Tokushima University, 2-1 Minamijosanjima-cho, Tokushima 770-8513, Japan

**Keywords:** *Paeonia lactiflora*, PC12 cells, NGF, isorhamnetin-3-*O*-glucoside, astragalin, neurite outgrowth-promoting activity

## Abstract

Isorhamnetin-3-*O*-glucoside and astragalin, flavonol glucosides, were isolated from the petals of *Paeonia lactiflora* as neurite outgrowth-promoting compounds. Isoquercitrin, formed by demethylating the B ring of isorhamnetin-3-*O*-glucoside or by adding a hydroxyl group to the B ring of astragalin, was evaluated for neurite outgrowth-promoting activity and was compared with the activities of isorhamnetin-3-*O*-glucoside and astragalin. The activities of isorhamnetin, kaempferol, and quercetin, aglycones corresponding to isorhamnetin-3-*O*-glucoside, astragalin, and isoquercitrin, respectively, were also evaluated. Isorhamnetin-3-*O*-glucoside and astragalin showed much stronger neurite outgrowth-promoting activities than the activities of the other tested flavonoids. They exhibited relatively weak anti-oxidant activities and moderate AChE inhibitory activities compared to the activities of the other tested flavonoids. Isorhamnetin-3-*O*-glucoside and astragalin promoted morphological neurite outgrowth and the expression of neurofilaments induced by NGF in PC12 cells. Isorhamnetin-3-*O*-glucoside and astragalin might be candidate compounds as neural differentiation agents in peripheral nerves and functional food ingredients preventing cognitive decline.

## 1. Introduction

Edible flowers are used as a garnish for food to improve the aesthetic appearance of the food. In addition, interest in edible flowers has been increasing in recent years since bioactive compounds contained in flowers could contribute to the improvement of health. Triterpene diols, triols, and their fatty acid esters derived from edible chrysanthemum (*Chrysanthemum morifolium*) flower extracts were reported to exhibit inhibitory activity against 12-*O*-tetradecanoylphorbol-13-acetate-induced inflammation [1]. Kaempferol glycosides from *Camellia nitidissima*, which is a medicinal and edible plant in China, are known to inhibit the formation of advanced glycation end products (AGEs) [2], and 1-*O*-(*E*)-caffeoyl-β-*D*-glucopyranoside found in cherry blossom flowers, which are used as a processed food soaked in salty vinegar, also inhibits the production of AGEs [3]. It was reported that mandelamide isolated from *Prunus persica* shows anti-adipogenic activity, suggesting that mandelamide possesses anti-obesity properties [4]. The flowers of *Paeonia lactiflora*, as well as chrysanthemum and cherry blossoms, are edible. *P. lactiflora* is a perennial plant that belongs to the Paeoniaceae family and grows wild in China and Eastern Asia [5]. *P. lactiflora* has a sweet and fresh fragrance and is widely cultivated as an ornamental plant. The roots of *P. lactiflora* are used for medicinal purposes since ancient times [6]. Paeoniflorin, a monoterpene glucoside contained in the roots [7], is known to have anti-hypoglycemic activity [7], anti-inflammatory activity [8], and protective activity against amyloid β-induced neurotoxicity [9]. However, the bioactive compounds derived from the flowers of *P. lactiflora*, which are used as herbal tea, have not been reported. 

Recently, food-derived ingredients were studied for their effects on cognitive function diseases such as Alzheimer’s disease. 6-Shogaol, isolated from the rhizome of ginger (*Zingiber officinale var. officinale*), shows neurite outgrowth activity comparable to that of nerve growth factor (NGF), which is related to memory and learning in the brain [10]. Isofuranodiene, isolated from celery (*Apium graveolens* L.) flowers, shows neurite outgrowth-promoting activity in the presence of NGF [11]. These compounds are believed to have the potential for beneficial preventive and therapeutic uses in neurodegenerative diseases. Since the flowers of *P. lactiflora* could be ingested on a daily basis by use as a herbal tea, the discovery of compounds that exhibit neurite outgrowth activity or neurite outgrowth-promoting activity from the flowers of *P. lactiflora* may contribute not only to the maintenance and promotion of health but also the prevention of cognitive dysfunction. This study was carried out to investigate the neurite outgrowth-promoting compounds from the flowers of *P. lactiflora* (one of the peony flowers) in PC12 cells. Herein, the isolation and identification of active compounds and their structure-activity relationships are reported. The results of the investigations of the anti-oxidant activity and acetylcholinesterase inhibitory activity are also reported.

## 2. Results and Discussion

### 2.1. Isolation of Compounds ***1*** and ***2*** from the Extract of the Petals of P. lactiflora

The petals of *P. lactiflora* (135.90 g) were extracted with MeOH/H_2_O (8/2, *v*/*v*), and the extract showed neurite outgrowth-promoting activity in the presence of nerve growth factor (NGF) or dibutyryl cyclic AMP (Bt_2_cAMP) in PC12 cells (Figure S1 in the supplementary materials). NGF binds to the tropomyosin receptor kinase A receptor and extends neurites via a signaling cascade that includes extracellular signal-regulated kinase (ERK) [12,13]. As another signal pathway, NGF increases intracellular cAMP concentrations, which induces neurite formation [14]. Bt_2_cAMP, which is a membrane-permeable cAMP derivative, is metabolized intracellularly to cAMP and shows a neurite formation effect. Therefore, in order to efficiently evaluate the neurite outgrowth-promoting activity in a short time and to purify the active compounds, Bt_2_cAMP was applied as a neurite formation inducer in PC12 cells. 

**Figure 1:**
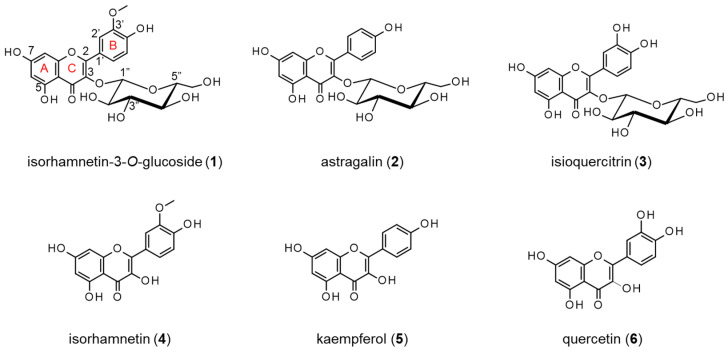
Chemical structures of compounds **1** (isorhamnetin-3-*O*-glucoside) and **2** (astragalin) isolated from the extracts of the petals of *P. lactiflora* and the analogs.

To investigate the neurite outgrowth-promoting compounds from the petals of *P. lactiflora*, a bioassay-guided purification was carried out. After the concentration of the extract, it was dissolved in H_2_O and partitioned with n-hexane, EtOAc, and water-saturated 1-butanol successively. Then, the EtOAc layer was chromatographed on a Diaion HP20 column and TOYOPEARL HW-40F twice via activity-guided fractionation to obtain active compounds **1** (2.0 mg) and **2** (10.7 mg). Compounds **1** and **2** were identified as isorhamnetin-3-*O*-glucoside and astragalin, respectively (Figure 1), with NMR (Figures S2–S4 and S6–S10 in the supplementary materials), MS, and HPLC analyses (Figures S5 and S11 in the supplementary materials). The two isolated flavonol glucosides are known compounds and have already been reported to be contained in the flowers of *P. lactiflora* [15]. Previously, isorhamnetin-3-*O*-glucoside was isolated from the leaves of *Sarracenia purpurea* as an anti-diabetic active compound [16,17] and was also shown to have anti-microbial activity [18]. Astragalin was isolated from the aerial part of *Orostachys japonicus* as a calpain inhibitor [19]. Moreover, astragalin was reported to show angiotensin-converting enzyme inhibitory activity [20] and glycation inhibitory activity [21]. In this study, two already-known compounds from the petals of *P. lactiflora* were isolated for the first time as neurite outgrowth-promoting compounds via an activity-guided purification.

### 2.2. Structure-Activity Relationships of Compounds ***1*** and ***2*** and Their Analogs

**Figure 2:**
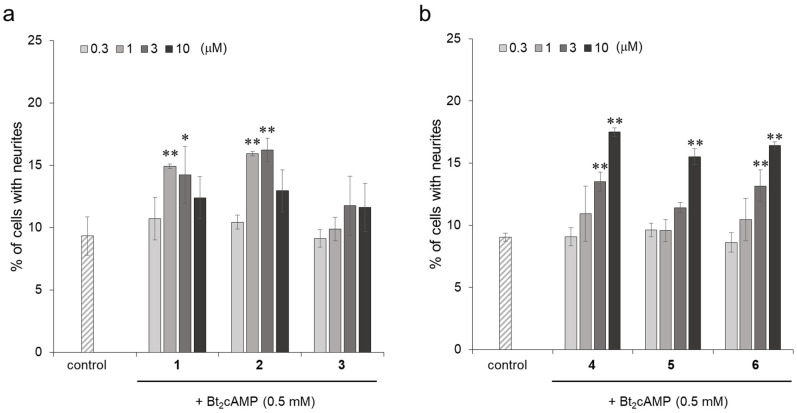
The neurite outgrowth-promoting activities of compounds **1**–**6** in the presence of Bt_2_cAMP in PC12 cells. (**a**) Promotion by flavonol glucosides; compounds **1**–**3** of neurite formation induced by Bt_2_cAMP in PC12 cells. (**b**) Promotion by flavonols; compounds **4**–**6** of neurite formation induced by Bt_2_cAMP in PC12 cells. PC12 cells were plated at 4.0 × 10^3^ cells/well and cultured with the flavonoids at 0.3, 1, 3, and 10 μM in the presence of 0.5 mM of Bt_2_cAMP. The extent of neurite outgrowth was measured at 24 h and is expressed as the mean percentage of 300-400 cells. The data represent the means ± standard deviations from three independent experiments. * *p* < 0.05, ** *p* < 0.01 (Dunnett’s test) as compared with the control (0.5 mM Bt_2_cAMP only).

In order to investigate the effect of the neurite outgrowth-promoting activity caused by the differences in chemical structures, the activities were evaluated and compared using compound **1**, compound **2**, isoquercitrin (**3**), isorhamnetin (**4**), kaempferol (**5**), and quercetin (**6**). Their chemical structures are shown in Figure 1. Compound **3** is a flavonol glucoside with hydroxyl groups at the 3′ and 4′ positions of the B ring. Compounds **4**, **5**, and **6** are aglycons of compounds **1**, **2**, and **3**, respectively. Compounds **4** and **6** are known flavonols with neurite outgrowth-promoting activities [22,23]. Among the flavonol glucosides, compounds **1** and **2** showed significant neurite outgrowth-promoting activities at concentrations of 1 and 3 μM and diminished activities at a concentration of 10 μM, while compound **3** did not show any activity at a concentration range from 0.3 to 10 μM (Figure 2a). The causes of the decreased activity at high concentrations of active compounds were not clarified, although similar profiles of activity were shown in other papers [24,25,26]. The flavonols showed neurite outgrowth-promoting activities in a concentration-dependent manner, and the intensities of their activities were similar (Figure 2b). Compounds **1** and **2**, flavonol glucosides, showed activities at lower concentrations than those of their aglycons, indicating that a glucose moiety at the C-3 position has the ability to enhance the activities of these flavonols. However, compound **3** with a glucose moiety at the C-3 position showed no activity at the tested concentrations. Compound **3** has the highest polarity of the B ring among the three flavonol glucosides since compound **3** is formed by demethylating the B ring of compound **1** or by adding a hydroxyl group to the B ring of compound **2**. It was suggested that a certain degree of low polarity of the B ring is important for flavonol glucosides to exert their neurite outgrowth-promoting activities. Compounds **1** and **2**, which have a glucose moiety at the C-3 position and B ring with a certain degree of low polarity, showed stronger neurite outgrowth-promoting activities than that of compounds **4** and **6**, which are known neurite outgrowth-promoting compounds. Therefore, it was revealed that the presence of not only glucose at the C-3 position but also a B ring with certain low polarity is important for showing strong neurite outgrowth-promoting activity.

### 2.3. 2,2-Diphenyl-1-Picrylhydrazyl (DPPH) Radical Scavenging Activities of Isorhamnetin-3-O-glucoside (***1***), Astragalin (***2***), and Their Analogs

**Figure 3:**
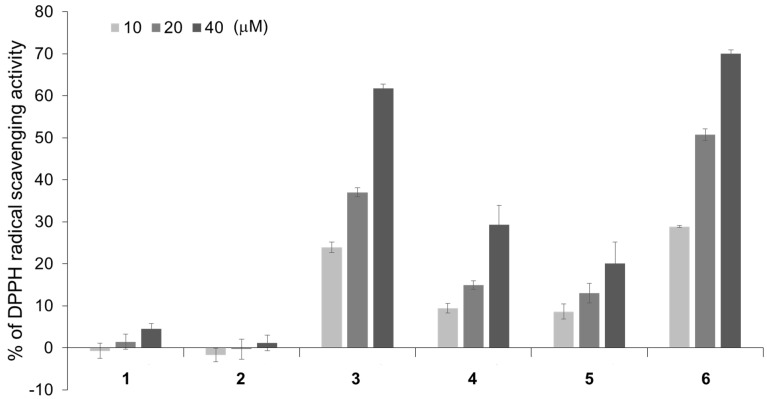
The anti-oxidant activities of compounds **1**–**6** based on a DPPH radical scavenging assay. The flavonoids were treated at concentrations of 10, 20, and 40 μM against 100 μM of DPPH radical. The data represent the means ± standard deviations from three independent experiments.

Many flavonoids are known to have anti-oxidant activities. Stimulation with NGF is thought to increase the concentration of reactive oxygen species (ROS) in neurons, and these ROS act as intracellular signal mediators to promote neurite differentiation [27]. Hence, the anti-oxidant activities of compounds **1** and **2**, which were isolated from the petals of *P. lactiflora* as neurite outgrowth-promoting compounds and their analogs were investigated. The anti-oxidant activities of compounds **1**–**6** were evaluated with a DPPH radical scavenging assay. The DPPH radical scavenging activity of quercetin (**6**) is well known [28]. Compound **3** showed anti-oxidant activity as intense as that of compound **6**, while compounds **4** and **5** showed weak anti-oxidant activities (Figure 3). Compounds **1** and **2**, which showed the strongest neurite outgrowth-promoting activities of the evaluated flavonoids, did not have much anti-oxidant activity. This result revealed that there is a negative correlation between the anti-oxidant activity and neurite outgrowth-promoting activity. Since compounds **1** and **2** showed relatively weak anti-oxidant activities, it is unlikely that they scavenge ROS, which are the intracellular signal mediators of neuronal differentiation with NGF stimulation, thus, exhibiting strong neurite outgrowth-promoting activities. Although some compounds, such as quercetin (**6**), exhibit neurite outgrowth-promoting activity while showing strong anti-oxidant activity, the results suggested that a weak anti-oxidant activity was important to show strong neurite outgrowth-promoting activity.

### 2.4. Acetylcholinesterase (AChE) Inhibitory Activities of Isorhamnetin-3-O-glucoside (***1***), Astragalin (***2***) and Their Analogs

**Figure 4:**
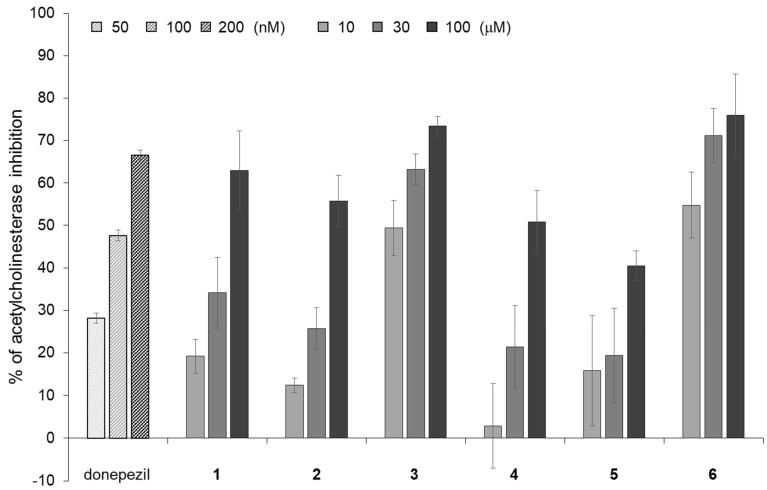
The AChE inhibitory activities of compounds **1**–**6**. The reaction mixtures consisted of 10, 30, and 100 μM of flavonoids, 0.1 U/mL of AchE, and 0.25 mM of 1-naphthyl acetate solution. Donepezil was used as a positive control and was assessed at concentrations of 50, 100, and 200 nM. After mixing for 90 s and incubating at 25 °C for 90 s, the reaction was stopped with a 5% solution of sodium dodecyl sulfate. The color was developed with the Fast Blue B solution, and the absorbance was measured at 620 nm. The data represent the means ± standard deviations from three independent experiments.

Since the AChE inhibitory activity, as well as the neurite outgrowth-promoting activity, are effective for cognitive dysfunctions, AChE inhibitors are used to alleviate symptoms in patients with cognitive dysfunctions [29]. It is thought that a decrease in the level of acetylcholine (ACh), a neurotransmitter associated with memory in the brain, leads to a decline in cognitive function. To prevent a decrease in the level of ACh, it is necessary to inhibit the function of AChE, which is an esterase that decomposes ACh. Since AChE inhibitors could lead to an increase in ACh levels and maintain cognitive function, they were recently used as symptomatic treatments for Alzheimer’s disease and other cognitive dysfunctions [29]. Donepezil is one of the AChE inhibitors used for the treatment of Alzheimer’s disease. Compounds **1** and **2**, which have strong neurite outgrowth-promoting activities, and their analogs, were evaluated for AChE inhibitory activity and compared with donepezil. Six flavonoids inhibited AChE activity at much higher concentrations than the concentrations of donepezil (Figure 4). Among the six flavonoids, compounds **3** and **6** showed strong inhibitory activities, and compounds **1** and **2** showed moderate inhibitory activities. Compounds **4** and **5** had weak inhibitory activities. The results revealed that there were different tendencies in the intensity of the neurite outgrowth-promoting activities and AChE inhibitory activities of the six flavonoids. Compounds **1** and **2** have not only strong neurite outgrowth-promoting activities but also moderate AChE inhibitory activities among the six flavonoids. Therefore, isorhamnetin-3-*O*-glucoside (**1**) and astragalin (**2**) may contribute to the prevention of cognitive dysfunction.

### 2.5. Promoting the Activity of Isorhamnetin-3-O-glucosides (***1***) and Astragalin (***2***) for Neurite Formation Induced by NGF in PC12 Cells

**Figure 5:**
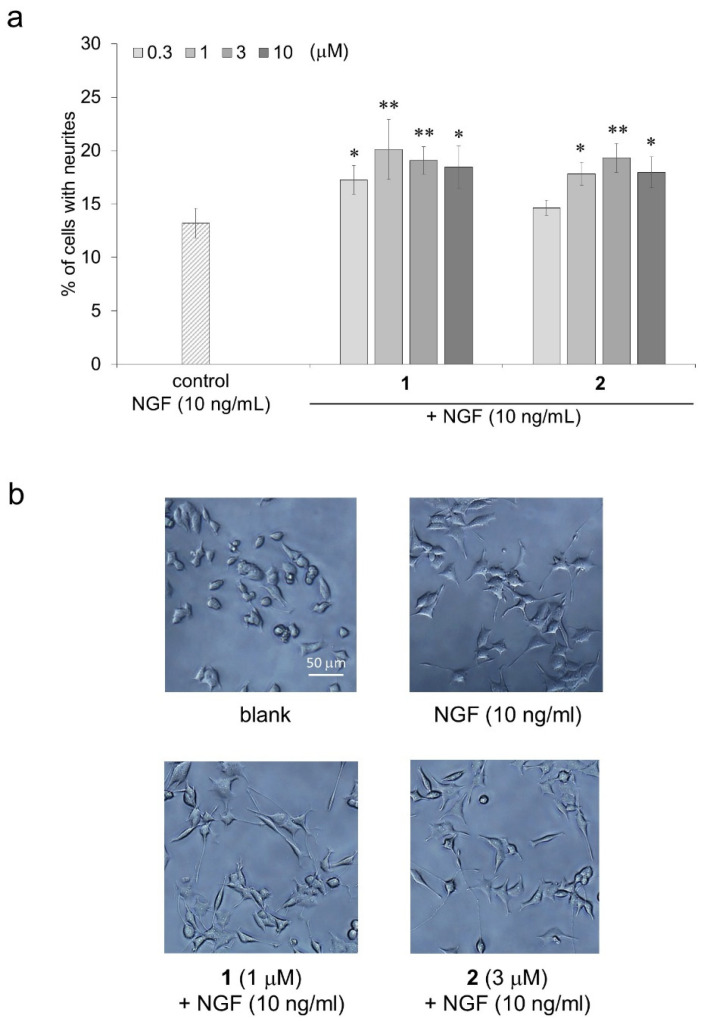
The neurite outgrowth-promoting activities of compounds **1** and **2** in the presence of NGF. (**a**) Promotion by compounds **1** and **2** of neurite formation induced by NGF in PC12 cells. PC12 cells were plated at 2.0 × 10^3^ cells/well and cultured with compounds **1** or **2** at 0.3–10 μM in the presence of 10 ng/mL of NGF. The extent of neurite outgrowth was measured at 48 h and is expressed as the mean percentage of 300–400 cells. The data represent the means ± standard deviations from three independent experiments. * *p* < 0.05, ** *p* < 0.01 (Dunnett’s test) as compared with the control (10 ng/mL of NGF). (**b**) The effects of compounds **1** and **2** on neurite outgrowth induced by NGF. PC12 cells were incubated for 48 h without NGF (blank), with NGF only (control), or with NGF and the flavonol glucosides. The final NGF concentration was 10 ng/mL, and the final concentrations of compounds **1** and **2** were 1 μM and 3 μM, respectively. Scale bar = 50 μm.

The neurite outgrowth-promoting activities of compounds **1** and **2**, which showed strong activities in the presence of Bt_2_cAMP in the structure-activity relationship investigation, were then evaluated in the presence of NGF. The neurite outgrowth-promoting activity of compounds **1** and **2** in the presence of NGF showed the same profile as that of their activity in the presence of Bt_2_cAMP (Figure 5a). The morphological appearances of the PC12 cells treated with compounds **1** or **2** in the presence of NGF are shown in Figure 5b. The results indicate that isorhamnetin-3-*O*-glucoside (**1**) and astragalin (**2**) could promote neurite outgrowth induced by NGF as well as Bt_2_cAMP. 

**Figure 6:**
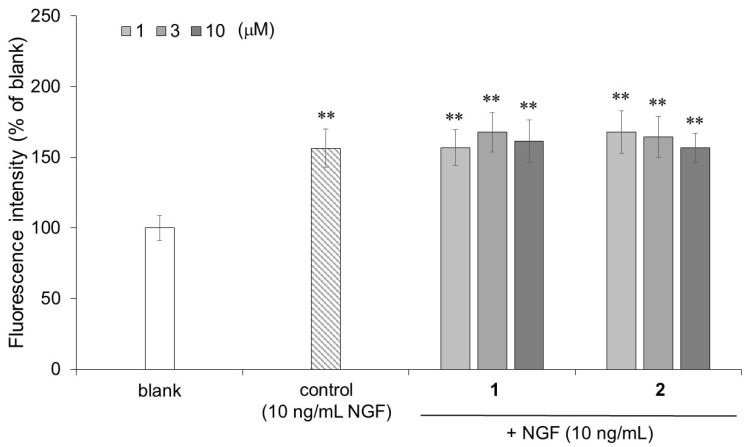
ELISA for the neurofilaments in the PC12 cells with differentiated neurites stimulated by compounds **1** or **2** in the presence of NGF. PC12 cells were plated at 2.0 × 10^3^ cells/well and incubated for 48 h without NGF (blank), with NGF only (control), or with NGF and compound **1** or **2** at 0.3–10 μM. An ELISA was performed with a rabbit anti-neurofilament antibody (1000:1) followed by treatment with peroxidase-conjugated goat anti-rabbit IgG (60,000:1). The bound enzyme activity was detected by reacting with ADHP, and the fluorescence intensity at 535 nm/590 nm of each well was measured. The data represent the means ± standard deviations from three independent experiments. ** *p* < 0.01 (Dunnett’s test) as compared with the blank.

Neurofilaments are type IV intermediate filaments that are specifically expressed in nerve cells and are involved in the maintenance of nerve thickness. Hence, investigating the increased expression of neurofilaments in PC12 cells was used as an indicator of neuronal differentiation. In the cell-ELISA, the PC12 cells stimulated by NGF (control) and compounds **1** and **2** in the presence of NGF showed significantly higher neurofilament expression than that of the untreated PC12 cells (blank) (Figure 6). The PC12 cells stimulated by 3 μM of compound **1** (167.7%) or 1 μM of compound **2** (167.8%) in the presence of NGF also tended to show slightly increased neurofilament expression compared to that of the PC12 cells stimulated by NGF only (control: 156.4%). Isorhamnetin-3-*O*-glucoside (**1**) and astragalin (**2**), which promote neurite outgrowth, also increased the neurofilament expression. 

The compounds promoting neurite outgrowth are thought to be effective for treating cognitive dysfunctions, such as Alzheimer’s disease, by helping the differentiation of neurons associated with memory and learning in the brain [30]. Isorhamnetin-3-*O*-glucoside (**1**) and astragalin (**2**) have strong neurite outgrowth-promoting activities; however, they are metabolized by human intestinal flora to create aglycons [31]. This fact suggests that the two flavonol glucosides cannot reach the brain and cannot be applied as a therapeutic agent for cognitive dysfunctions. On the other hand, previous studies have shown that NGF expression is induced from fibroblasts, mast cells, and Schwann cells in the tissues damaged by rheumatoid arthritis, cystitis, and prostatitis [32,33,34]. It is believed that the induced NGF expression activates sensory nerves innervating the damaged tissues and is involved in the repair of the damaged area [35]. Hence, isorhamnetin-3-*O*-glucoside (**1**) and astragalin (**2**) are potential nerve differentiation enhancers for damaged peripheral nerves. In addition, even when isorhamnetin-3-*O*-glucoside (**1**) and astragalin (**2**) are metabolized to isorhamnetin (**4**) and kaempferol (**5**), isorhamnetin (**4**) and kaempferol (**5**) could exhibit neurite outgrowth-promoting activity, although the activity is weaker than that of these glucosides. Isorhamnetin (**4**) and kaempferol (**5**) were reported to be able to cross the blood-brain barrier [36], suggesting that they could exert neurite outgrowth-promoting activity in the brain to prevent cognitive dysfunctions. Furthermore, it was reported that the oral administration of quercetin (**6**), a structural analog of isorhamnetin (**4**) and kaempferol (**5**), tended to improve memory and cognitive function in a mouse model of Alzheimer’s disease [37]. Since the flowers of *P. lactiflora* containing isorhamnetin-3-*O*-glucoside (**1**) and astragalin (**2**) are used in herbal tea, those compounds could be ingested on a daily basis. Therefore, isorhamnetin-3-*O*-glucoside (**1**) and astragalin (**2**) may have potential applications not only as functional food ingredients for preventing cognitive decline but also as neural differentiation agents in peripheral nerves.

## 3. Materials and Methods

### 3.1. Chemicals

All of the solvents for the extraction and chromatography were commercially purchased. Diaion HP20 (Mitsubishi Chemical Corporation, Tokyo, Japan) and TOYOPEARL HW-40F (Tosoh Corporation, Tokyo, Japan) were utilized for column chromatography. Isorhamnetin-3-*O*-glucoside (ChromaDex Inc., Los Angeles, CA, USA), astragalin, isoquercitrin, kaempferol (Cayman Chemical Company, Ann Arbor, MI, USA), isorhamnetin (Tokyo Chemical Industry Corporation, Tokyo, Japan), and quercetin (Sigma-Aldrich Japan, Tokyo, Japan) were used as standard samples to evaluate the neurite outgrowth-promoting activity, anti-oxidant activity and acetylcholinesterase (AChE) inhibitory activity. Dibutyryl cyclic AMP (Bt_2_cAMP) (Sigma-Aldrich, Japan) and recombinant rat β-NGF (NGF) (R&D SYSTEMS, Minneapolis, MN, USA) were used as inducers of neuronal differentiation. 2,2-Diphenyl-1-picrylhydrazyl (DPPH) radical (Sigma-Aldrich Japan) was used for the evaluation of the anti-oxidant activity. Acetylcholinesterase (AChE) from *Electrophorus electricus* (Sigma-Aldrich Japan), 1-naphthyl acetate (Tokyo Chemical Industry Corporation) and Fast Blue B salt (MP Biomedicals, Irvine, CA, USA) were used for the evaluation of the AChE inhibitory activity. An anti-neurofilament 200 antibody produced in rabbits, anti-rabbit IgG (whole molecule)-peroxidase antibody produced in goats, H_2_O_2_ (Sigma-Aldrich Japan), paraformaldehyde, Tween 20 (FUJIFILM Wako Pure Chemical Corporation, Osaka, Japan), and 10-Acetyl-3,7-dihydroxyphenoxazine (ADHP) (Cayman Chemical Company) were used for the cell-ELISA.

### 3.2. Instruments

The NMR spectra were recorded using a Varian NMR System 600 MHz with CD_3_OD. The values of the chemical shifts are expressed in ppm, and each coupling constant (*J*) is expressed in Hz. The electron spray ionization (ESI) high-resolution mass spectra were obtained on a Bruker Daltonics MicrOTOF II instrument using direct sample injection. The Cell-ELISA and DPPH radical scavenging assay were performed on an Infinite 200 Pro M Nano+ (Tecan Japan Corporation, Tokyo, Japan), and AChE inhibitory activity was measured on a Multiskan FC (Thermo Fisher Scientific K.K., Tokyo, Japan).

### 3.3. Extraction and Isolation

The petals of *Paeonia lactiflora* Pall. were collected in May 2018 from the Field Science Center, Prefectural University of Hiroshima, Japan. The fresh petals of *P. lactiflora* (135.90 g, fr. wt.) were extracted with 1360 mL of MeOH/H_2_O (80/20, *v*/*v*) at room temperature for 3 days, and then the extracts were evaporated to become dry (19.0 g, dry wt.). The extracts were dissolved in 750 mL of H_2_O and partitioned with n-hexane (750 mL, twice), EtOAc (750 mL, twice), and water-saturated 1-butanol (375 mL, twice) in that order, and the separated layers were evaporated to become dry (hexane layer: 20.3 mg, EtOAc layer: 5.94 g, water-saturated 1-butanol layer: 1.21 g, and water layer: 7.82 g). The EtOAc layer, which showed neurite outgrowth-promoting activity, was applied to a Diaion HP20 column (5.0 cm i.d. × 41.0 cm) and eluted with a stepwise MeOH/H_2_O gradient (60/40, 65/35, 70/30, 75/25, 80/20, *v*/*v*, 800 mL each). The eluted fractions in MeOH/H_2_O (65/35, *v*/*v*) were combined, and the combined fraction showed neurite outgrowth-promoting activity. Moreover, the combined fraction (72.4 mg) was purified by using a TOYOPEARL HW-40F (1.5 cm i.d. × 68.0 cm) with 420 mL of MeOH/H_2_O (60/40, *v*/*v*) to obtain 175 fractions. Fractions 90–115 were combined, and the combined fraction showed activity. The combined active fraction (35.6 mg) was chromatographed on TOYOPEARL HW-40F (1.5 cm i.d. × 67.0 cm) again and eluted with 300 mL of MeOH/H_2_O/AcOH (60/39/1, *v*/*v*/*v*) to obtain 150 fractions. Fractions 85–90 (compound **1**, 2.0 mg) and 99–103 (compound **2**, 10.7 mg), which showed significant activity, were isolated as yellow powders, respectively.

### 3.4. Spectroscopic Data of Compounds

Compound **1** (isorhamnetin-3-*O*-glucoside): Yellow powder. HRMS: *m*/*z* 477.1031 [M-H]^−^ (calcd. for C_22_H_21_O_12_, 477.1038). ^1^H-NMR (600 MHz, OD_3_OD): δ 7.94 (1H, d, *J* = 2.4 Hz, H-2′), 7.58 (1H, dd, *J* = 8.1 Hz and 2.1 Hz, H-6′), 6.90 (1H, d, *J* = 8.1 Hz, H-5′), 6.40 (1H, d, *J* = 2.1 Hz, H-8), 6.20 (1H, d, *J* = 2.1 Hz, H-6), 5.43 (1H, d, *J* = 7.8 Hz, H-1′′), 3.94 (3H, s, OMe), 3.73 (1H, dd, *J* = 12.0 Hz and 2.4 Hz, H-6′′a), 3.56 (1H, dd, *J* = 12.0 Hz and 6.0 Hz, H-6′′b), 3.47-3.42 (2H, m, H-2′′ and H-3′′), 3.32-3.29 (overlapped with solvent, H-4′′), 3.23 (1H, m, H-5”). These ^1^H-NMR data were consistent with those of isorhamnetin-3-*O*-glucoside in another report [38]. Also, compound **1** was identified as isorhamnetin-3-*O*-glucoside with HPLC co-chromatography with the standard. 

Compound **2** (astragalin): Yellow powder. HRMS: *m*/*z* 447.0926 [M-H]^−^ (calcd. for C_21_H_19_O_11_, 447.0933). ^1^H-NMR (600 MHz, OD_3_OD): δ 8.06 (2H, dd, *J* = 6.9 Hz and 2.1 Hz, H-2′, 6′), 6.88 (2H, dd, *J* = 6.9 Hz and 2.1 Hz, H-3′, 5′), 6.40 (1H, d, *J* = 2.1 Hz, H-8), 6.20 (1H, d, *J* = 2.1 Hz, H-6), 5.27 (1H, d, *J* = 7.8 Hz, H-1′′), 3.69 (1H, dd, *J* = 12.0 Hz and 2.4 Hz, H-6′′a), 3.52 (1H, dd, *J* = 12.0 Hz and 5.4 Hz, H-6′′b), 3.44 (1H, t, *J* = 9.0 Hz, H-2′′), 3.41 (1H, t, *J* = 9.0 Hz, H-3′′), 3.32-3.29 (overlapped with solvent, H-4′′), 3.21-3.18 (1H, m, H-5′′). ^13^C-NMR (150 MHz, CD_3_OD): δ 178.1 (C-4), 164.6 (C-7), 161.7 (C-5 or C-9), 160.2 (C-4’), 157.6 (C-2), 157.1 (C-5 or C-9), 134.0 (C-3), 130.9 (C-2′, 6′), 121.3 (C-1′), 114.6 (C-3′, 5′), 104.3 (C-10), 102.5 (C-1′′), 98.4 (C-6 or C-8), 93.3 (C-6 or C-8), 77.0 (C-5′′), 76.6 (C-3′′), 74.3 (C-2′′), 69.9 (C-4′′), 61.2 (C-6′′). These NMR data were consistent with those of astragalin in another report [39]. Also, compound **2** was identified as astragalin with HPLC co-chromatography with the standard.

### 3.5. Cell Culture

PC12 cells were purchased from RIKEN BRC Cell Bank (Tsukuba, Japan). The cells were grown in RPMI-1640 supplemented with 10% HS (Lot. 1517707, Gibco, Waltham, MA, USA), 5% FBS (Lot. 42F9155K, Gibco), 100 U/mL penicillin G, and 100 mg/mL streptomycin at 37 °C in a humidified atmosphere of 95% air/5% CO_2_. The medium was changed every one or two days.

### 3.6. Neurite Outgrowth-Promoting Activity

The PC12 cells from the stock culture were suspended in the medium and plated at 4.0 × 10^3^ cells/90 μL/well (for the evaluation in the presence of Bt_2_cAMP) or 2.0 × 10^3^ cells/90 μL/well (for the evaluation in the presence of NGF) in 96-well plates (Thermo Fisher Scientific K.K., Tokyo, Japan) with Cell-matrix type I–P (collagen) (Nitta Gelatin Inc., Osaka, Japan) and incubated in a humidified atmosphere of 5% CO_2_ at 37 °C. After 24 h, 5 μL of Bt_2_cAMP at 10 mM (final concentration: 0.5 mM) or NGF at 200 ng/mL (final concentration: 10 ng/mL), and 5 μL of each sample or the medium only (control) were added to the culture medium (the final concentration of each sample is indicated in the figures). At 24 h after the addition of Bt_2_cAMP and the samples or at 48 h after the addition of NGF and the samples, the medium was aspirated, and the PC12 cells were fixed with a phosphate buffer (pH 7.2, 100 mM) containing 1% glutaraldehyde and stained with a Giemsa stain solution. Then the 96-well plates were washed twice with Milli-Q grade water. The number of cells bearing neurites longer than twice the diameter of one cell body after treatment was divided by the total number of cells, which was 300–400 cells per well.

### 3.7. 2,2-Diphenyl-1-Picrylhydrazyl (DPPH) Radical Scavenging Assay

The DPPH radical scavenging activity was evaluated by modifying the method previously reported [40]. The DPPH radical scavenging assay was carried out in a 96-well plate. Flavonoids dissolved with 160 μL of 50% citrate buffer (10 mM, pH 6.0)/50% ethanol (the final concentrations of flavonoids were 10, 20, and 40 μM) or 160 μL of 50% citrate buffer (10 mM, pH 6.0)/50% ethanol as a control sample were added to 40 μL of freshly prepared DPPH radical dissolved with ethanol (final concentration of DPPH: 100 μM). The plate was incubated in the dark for 1 h at room temperature. The absorbance was measured at 524 nm using a microplate reader.

### 3.8. Acetylcholinesterase (AChE) Inhibitory Activity

The AChE inhibitory activity was evaluated by modifying the method previously reported [41]. The AChE inhibition assay was performed using a spectrophotometric microplate assay. Donepezil, an AChE inhibitor used for the treatment of dementia, was evaluated as a positive control in this assay. The reaction mixture consisted of 65 μL of flavonoids (final concentrations of 10, 30, and 100 μM containing 1% DMSO) or donepezil (final concentrations of 50, 100, and 200 nM containing 1% DMSO) dissolved with a phosphate buffer and 65 μL of a solution of AChE (final concentration of 0.1 U/mL in 100 mM phosphate buffer pH 7.4). The enzymatic reaction was initiated by the addition of 130 μL of 0.5 mM 1-naphthyl acetate dissolved with a phosphate buffer containing 1% DMSO. After mixing for 90 s and incubating at 25 °C for 90 s, the reaction was stopped with 20 μL of 5% sodium dodecyl sulfate. The color was developed with 20 μL of Fast Blue B solution (final concentration of 0.17 mM in H_2_O). The enzyme activity inhibition was quantified by the determination of the absorbance at 620 nm after the formation of the purple-colored diazonium dye as a percentage. The control sample consisted of the same amount of phosphate buffer containing a 1% DMSO solution instead of the flavonoids.

### 3.9. Detection of Neurofilaments in PC12 Cells with Cell-ELISA

The PC12 cells from the stock cultures were suspended in the medium and plated at 2.0 × 10^3^ cells/90 μL/well in 96-well plates that were pre-coated with collagen and incubated in a humidified atmosphere of 5% CO_2_ at 37 °C for 24 h. Then, 5 μL of isorhamnetin-3-*O*-glucoside or astragalin at a concentration of 20, 60, or 200 μM (final concentrations: 1, 3, and 10 μM) and 5 μL of NGF at a concentration of 200 ng/mL (final concentration of 10 ng/mL) were added to the culture medium. The cells were incubated in a humidified atmosphere of 5% CO_2_ at 37 °C for 48 h. Then, the cells cultured on the 96-well plates were fixed with 100 μL of 4% paraformaldehyde in a phosphate buffer (pH 7.4, 100 mM) for 30 min at room temperature. After the 4% paraformaldehyde was removed and the cells were washed three times with 200 μL of phosphate buffer saline (PBS(-)) (pH 7.4), the fixed cells were permeabilized with 50 μL of 0.2% Triton X-100/PBS(-) for 5 min at room temperature. The permeabilized cells were blocked with 200 μL of 2.5% BSA/PBS(-) for 1 h at room temperature after the cells were washed three times with 200 μL of PBS(-). After the removal of the 2.5% BSA/PBS(-), 50 μL of anti-neurofilament 200 antibody produced in rabbits (primary antibody) in 2.5% BSA/PBS(-) (1/1000, *v*/*v*) was added to each well and incubated overnight at 4 °C. After the primary antibody solution was removed and the cells were washed three times with 200 μL of 0.05% Tween 20/PBS(-), 50 μL of anti-rabbit IgG (whole molecule)-peroxidase antibody produced in goats (secondary antibody) in blocking solution (1/30,000, *v*/*v*) was added to each well and incubated for 1 h at room temperature. Then, the secondary antibody solution was removed, and the cells were washed three times with 0.05% Tween 20/PBS(-). The bound enzyme activity was detected by reacting with 10-acetyl-3,7-dihydroxyphenoxazine (ADHP) at room temperature in the dark. A total of 100 μL of ADHP solution contained 50 μM of ADHP and 0.01% H_2_O_2_ in citrate buffer (pH 5.0). The fluorescence in each well was then measured using a microplate reader at Ex./Em. = 535/590 nm.

## Data Availability

Not applicable.

## Supplementary Materials

The following supporting information can be downloaded at: https://www.mdpi.com/article/10.3390/molecules27227670/s1, Figure S1: Neurite outgrowth-promoting activity of the extract of the petals from *Paeonia lactiflora* in the presence of Bt_2_cAMP or NGF in PC12 cells.; Figure S2: ^1^H-NMR spectrum of compound **1** (isorhamnetin-3-*O*-glucoside); Figure S3: ^1^H-^1^H COSY spectrum of compound **1** (isorhamnetin-3-*O*-glucoside); Figure S4: NOESY spectrum of compound **1** (isorhamnetin-3-*O*-glucoside); Figure S5: HPLC analyses of compound **1** (isorhamnetin-3-*O*-glucoside); Figure S6: ^1^H-NMR spectrum of compound **2** (astragalin); Figure S7: ^13^C-NMR spectrum of compound **2** (astragalin); Figure S8: ^1^H-^1^H COSY spectrum of compound **2** (astragalin); Figure S9: HSQC spectrum of compound **2** (astragalin); Figure S10: HMBC spectrum of compound **2** (astragalin) Figure S11: HPLC analyses of compound **2** (astragalin).

## References

[1] Ukiya M., Akihisa T., Yasukawa K., Kasahara Y., Kimura Y., Koike K., Nikaido T., Takido M. (2001). Constituents of compositae plants. 2. Triterpene diols, triols, and their 3-*O*-fatty acid esters from edible chrysanthemum flower extract and their anti-inflammatory effects. J. Agric. Food Chem..

[2] Yang R., Wang W.X., Chen H.J., He Z.C., Jia A.Q. (2018). The inhibition of advanced glycation end-products by five fractions and three main flavonoids from *Camellia nitidissima* Chi flowers. J. Food Drug Anal..

[3] Shimoda H., Nakamura S., Morioka M., Tanaka J., Matsuda H., Yoshikawa M. (2011). Effect of cinnamoyl and flavonol glucosides derived from cherry blossom flowers on the production of advanced glycation end products (AGEs) and AGE-induced fibroblast apoptosis. Phytother. Res..

[4] Lee D., Kim J.Y., Qi Y., Park S., Lee H.L., Yamabe N., Kim H., Jang D.S., Kang K.S. (2021). Phytochemicals from the flowers of *Prunus persica* (L.) Batsch: Anti-adipogenic effect of mandelamide on 3T3-L1 preadipocytes. Bioorg. Med. Chem. Lett..

[5] Sang T., Crawford D., Stuessy T. (1997). Chloroplast DNA phylogeny, reticulate evolution, and biogeography of *Paeonia* (Paeoniaceae). Am. J. Bot..

[6] He D.Y., Dai S.M. (2011). Anti-inflammatory and immunomodulatory effects of *Paeonia lactiflora* pall., a traditional Chinese herbal medicine. Front. Pharm..

[7] Hsu F.L., Lai C.W., Cheng J.T. (1997). Antihyperglycemic effects of paeoniflorin and 8-debenzoylpaeoniflorin, glucosides from the root of *Paeonia lactiflora*. Planta Med..

[8] Zhu X., Fang Z.H. (2014). New monoterpene glycosides from the root cortex of *Paeonia suffruticosa* and their potential anti-inflammatory activity. Nat. Prod. Res..

[9] Zhong S.Z., Ge Q.H., Li Q., Qu R., Ma S.P. (2009). Peoniflorin attentuates Aβ_(1-42)_-mediated neurotoxicity by regulating calcium homeostasis and ameliorating oxidative stress in hippocampus of rats. J. Neurol. Sci..

[10] Seow S.L.S., Hong S.L., Lee G.S., Malek S.N.A., Sabaratnam V. (2017). 6-Shogaol, a neuroactive compound of ginger (*jahe gajah*) induced neuritogenic activity via NGF responsive pathways in PC-12 cells. BMC Complement. Altern. Med..

[11] Mustafa A.M., Maggi F., Papa F., Kaya E., Dikmen M., Öztürk Y. (2016). Isofuranodiene: A neuritogenic compound isolated from wild celery (*Smyrnium olusatrum* L., Apiaceae). Food Chem..

[12] Duan L., Hope J.M., Guo S., Ong Q., François A., Kaplan L., Scherrer G., Cui B. (2018). Optical activation of trkA signaling. Acs Synth. Biol..

[13] Vaudry D., Stork P.J., Lazarovici P., Eiden L.E. (2002). Signaling pathways for PC12 cell differentiation: Making the right connections. Science.

[14] Ravni A., Vaudry D., Gerdin M.J., Eiden M.V., Falluel-Morel A., Gonzalez B.J., Vaudry H., Eiden L.E. (2008). A cAMP-dependent, protein kinase A-independent signaling pathway mediating neuritogenesis through Egr1 in PC12 cells. Mol. Pharm..

[15] Shu X., Duan W., Liu F., Shi X., Geng Y., Wang X., Yang B. (2014). Preparative separation of polyphenols from the flowers of *Paeonia lactiflora* Pall. by high-speed counter-current chromatography. J. Chromatogr. B.

[16] Muhammad A., Guerrero-Analco J.A., Martineau L.C., Musallam L., Madiraju P., Nachar A., Saleem A., Haddad P.S., Arnason J.T. (2012). Antidiabetic compounds from *Sarracenia purpurea* used traditionally by the Eeyou Istchee Cree First Nation. J. Nat. Prod..

[17] Kong C.S., Seo Y. (2012). Antiadipogenic activity of isorhamnetin 3-*O*-β-D-glucopyranoside from *Salicornia herbacea*. Immunopharmacol. Immunotoxicol..

[18] Liu H., Mou Y., Zhao J., Wang J., Zhou L., Wang M., Wang D., Han J., Yu Z., Yang F. (2010). Flavonoids from *Halostachys caspica* and their antimicrobial and antioxidant activities. Molecules.

[19] Je Ma C., Jung W.J., Lee K.Y., Kim Y.C., Sung S.H. (2009). Calpain inhibitory flavonoids isolated from *Orostachys japonicus*. J. Enzym. Inhib. Med. Chem..

[20] Kameda K., Takaku T., Okuda H., Kimura Y., Okuda T., Hatano T., Agata I., Arichi S. (1987). Inhibitory effects of various flavonoids isolated from leaves of persimmon on angiotensin-converting enzyme activity. J. Nat. Prod..

[21] Kim H.Y., Moon B.H., Lee H.J., Choi D.H. (2004). Flavonol glycosides from the leaves of *Eucommia ulmoides* O. with glycation inhibitory activity. J. Ethnopharmacol..

[22] Xu S.L., Choi R.C., Zhu K.Y., Leung K.W., Guo A.J., Bi D., Xu H., Lau D.T., Dong T.T., Tsim K.W. (2012). Isorhamnetin, a flavonol aglycone from *Ginkgo biloba* L., induces neuronal differentiation of cultured PC12 cells: Potentiating the effect of nerve growth factor. Evid. Based Complement. Altern. Med..

[23] Chan G.K.L., Hu W.W.H., Zheng Z.X., Huang M., Lin Y.X.Y., Wang C.Y., Gong A.G.W., Yang X.Y., Tsim K.W.K., Dong T.T.X. (2018). Quercetin potentiates the NGF-induced effects in cultured PC 12 cells: Identification by HerboChips showing a binding with NGF. Evid. Based Complement. Altern. Med..

[24] Eik L.F., Naidu M., David P., Wong K.H., Tan Y.S., Sabaratnam V. (2012). *Lignosus rhinocerus* (Cooke) ryvarden: A medicinal mushroom that stimulates neurite outgrowth in PC-12 cells. Evid. Based Complement. Altern. Med..

[25] Tai A., Aburada M., Ito H. (2014). A simple efficient synthesis and biological evaluation of 3-*O*-methylascorbic acid. Biosci. Biotechnol. Biochem..

[26] Zhou X., Tai A., Yamamoto I. (2003). Enhancement of neurite outgrowth in PC12 cells stimulated with cyclic AMP and NGF by 6-acylated ascorbic acid 2-*O*-α-glucosides (6-Acyl-AA-2G), novel lipophilic ascorbate derivatives. Biol. Pharm. Bull..

[27] Suzukawa K., Miura K., Mitsushita J., Resau J., Hirose K., Crystal R., Kamata T. (2000). Nerve growth factor-induced neuronal differentiation requires generation of Rac1-regulated reactive oxygen species. J. Biol. Chem..

[28] Haraguchi H., Ishikawa H., Sanchez Y., Ogura T., Kubo Y., Kubo I. (1997). Antioxidative constituents in *Heterotheca inuloides*. Bioorg. Med. Chem..

[29] Arya A., Chahal R., Rao R., Rahman M.H., Kaushik D., Akhtar M.F., Saleem A., Khalifa S.M.A., El-Seedi H.R., Kamel M. (2021). Acetylcholinesterase inhibitory potential of various sesquiterpene analogues for Alzheimer’s disease therapy. Biomolecules.

[30] Fukuyama Y., Kubo M., Harada K. (2020). The search for, and chemistry and mechanism of, neurotrophic natural products. J. Nat. Med..

[31] Du L.Y., Zhao M., Xu J., Qian D.W., Jiang S., Shang E.X., Guo J.M., Duan J.A. (2014). Analysis of the metabolites of isorhamnetin 3-*O*-glucoside produced by human intestinal flora in vitro by applying ultraperformance liquid chromatography/quadrupole time-of-flight mass spectrometry. J. Agric. Food Chem..

[32] Aloe L., Tuveri M.A., Carcassi U., Levi-Montalcini R. (1992). Nerve growth factor in the synovial fluid of patients with chronic arthritis. Arthritis Rheum..

[33] Lowe E.M., Anand P., Terenghi G., Williams-Chestnut R.E., Sinicropi D.V., Osborne J.L. (1997). Increased nerve growth factor levels in the urinary bladder of women with idiopathic sensory urgency and interstitial cystitis. Br. J. Urol..

[34] Miller L.J., Fischer K.A., Goralnick S.J., Litt M., Burleson J.A., Albertsen P., Kreutzer D.L. (2002). Nerve growth factor and chronic prostatitis/chronic pelvic pain syndrome. Urology.

[35] Wu C., Boustany L., Liang H., Brennan T.J. (2007). Nerve growth factor expression after plantar incision in the rat. Anesthesiology.

[36] Rangel-Ordonez L., Noldner M., Schubert-Zsilavecz M., Wurglics M. (2010). Plasma levels and distribution of flavonoids in rat brain after single and repeated doses of standardized *Ginkgo biloba* Extract EGb 761. Planta Med..

[37] Paula P.C., Angelica Maria S.G., Luis C.H., Gloria Patricia C.G. (2019). Preventive effect of quercetin in a triple transgenic Alzheimer’s disease mice model. Molecules.

[38] Kim A.R., Jin Q., Jin H.G., Ko H.J., Woo E.R. (2014). Phenolic compounds with IL-6 inhibitory activity from *Aster yomena*. Arch. Pharm. Res..

[39] Han S., Hanh Nguyen T.T., Hur J., Kim N.M., Kim S.B., Hwang K.H., Moon Y.H., Kang C., Chung B., Kim Y.M. (2017). Synthesis and characterization of novel astragalin galactosides using β-galactosidase from *Bacillus circulans*. Enzym. Microb. Technol..

[40] Doi N., Togari H., Minagi K., Iwaoka Y., Tai A., Nakaoji K., Hamada K., Tatsuka M. (2021). 2-*O*-Octadecylascorbic acid represses RhoGDIβ expression and ameliorates DNA damage-induced abnormal spindle orientations. J. Cell. Biochem..

[41] Olennikov D.N., Kashchenko N.I., Chirikova N.K., Akobirshoeva A., Zilfikarov I.N., Vennos C. (2017). Isorhamnetin and quercetin derivatives as anti-acetylcholinesterase principles of marigold (*Calendula officinalis*) flowers and preparations. Int. J. Mol. Sci..

