# Supplementary material for: Neurite Outgrowth-Promoting Compounds from the Petals of Paeonia lactiflora in PC12 Cells
Source: Molecules. 2022 Nov 8;27(22):7670. doi: 10.3390/molecules27227670 (PMC9692541; doi:10.3390/molecules27227670)
Supplement: Supplementary file 1 [file molecules-27-07670-s001.zip › molecules-1967987-supplementary.pdf]

Supplementary Materials

Neurite Outgrowth-Promoting Compounds from Petals of *Paeonia lactiflora* in PC12 Cells

Takeru Koga[1], Hideyuki Ito[2], Yuji Iwaoka[2], Toshiro Noshita[3,4], Akihiro Tai[3,5,*]

[1] *Graduate School of Advanced Technology and Science, Tokushima University, 2-1 Minamijosanjima-cho, Tokushima 770-8506, Japan*

[2] *Faculty of Health and Welfare Science, Okayama Prefectural University, 111 Kuboki, Soja, Okayama 719-1197, Japan*

[3] *Faculty of Life and Environmental Sciences, Prefectural University of Hiroshima, 5562 Nanatsuka-cho, Shobara, Hiroshima 727-0023, Japan*

[4] *Department of Pharmacy, Gifu University of Medical Science, 4-3-3 Nijigaoka, Kani, Gifu 509-0293, Japan*

[5] *Graduate School of Technology, Industrial and Social Sciences, Tokushima University, 2-1 Minamijosanjima-cho, Tokushima 770-8513, Japan*

*** Corresponding author**

E-mail address: atai@tokushima-u.ac.jp

Contents:

Figure S1. Neurite outgrowth-promoting activity of the extract of the petals from *Paeonia lactiflora* in the presence of Bt_2cAMP or NGF in PC12 cells.

Figure S2. ^1H-NMR spectrum of compound **1** (isorhamnetin-3-*O*-glucoside)

Figure S3. ^1H-^1H COSY spectrum of compound **1** (isorhamnetin-3-*O*-glucoside)

Figure S4. NOESY spectrum of compound **1** (isorhamnetin-3-*O*-glucoside)

Figure S5. HPLC analyses of compound **1** (isorhamnetin-3-*O*-glucoside)

Figure S6. ^1H-NMR spectrum of compound **2** (astragalin)

Figure S7. ^{13}C-NMR spectrum of compound **2** (astragalin)

Figure S8. ^1H-^1H COSY spectrum of compound **2** (astragalin)

Figure S9. HSQC spectrum of compound **2** (astragalin)

Figure S10. HMBC spectrum of compound **2** (astragalin)

Figure S11. HPLC analyses of compound **2** (astragalin)

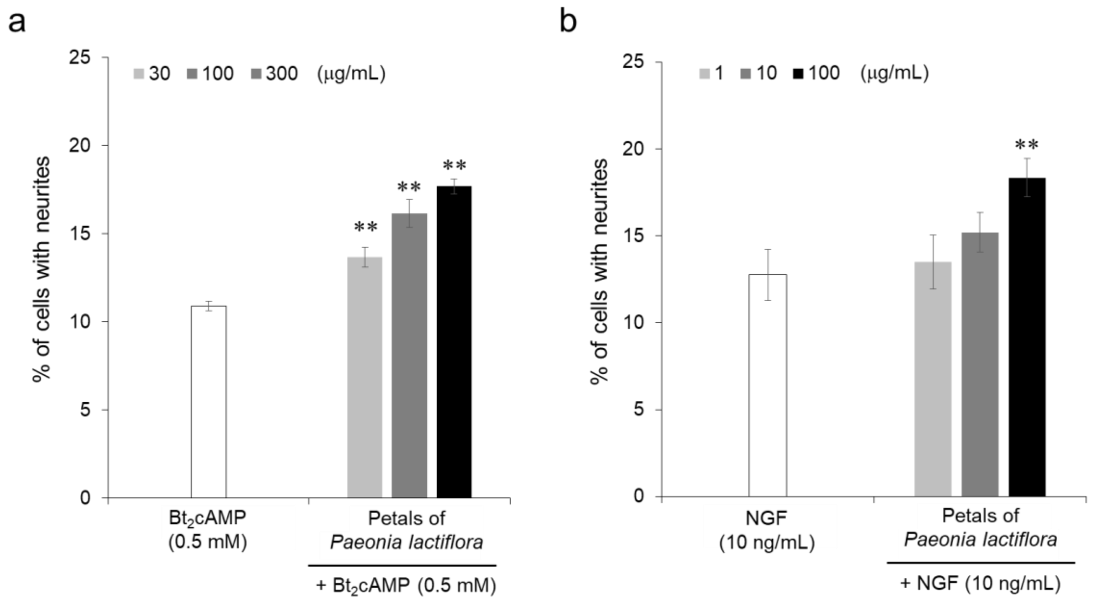

Figure S1. Neurite outgrowth-promoting activity of the extract of the petals from *Paeonia lactiflora* in the presence of Bt_2cAMP or NGF in PC12 cells. (a) Promotion by the extract of the petals from *Paeonia lactiflora* of neurite formation induced by Bt_2cAMP in PC12 cells. (b) Promotion by the extract of the petals from *Paeonia lactiflora* of neurite formation induced by NGF in PC12 cells. PC12 cells were plated at 4.0×10^3 cells/well and cultured with the extract at 30, 100, and 300 μg/mL in the presence of 0.5 mM of Bt_2cAMP or with the extract 1, 10 and 100 μg/mL in the presence of NGF. The extent of neurite outgrowth was measured at 24 h after the addition of Bt_2cAMP or at 48 h after the addition of NGF and is expressed as the mean percentage of 300-400 cells. The data represent means ± standard deviation from triplicate cultures. **p<0.01 (Dunnett's test) as compared with the control (0.5 mM Bt_2cAMP only or 10 ng/mL NGF only).

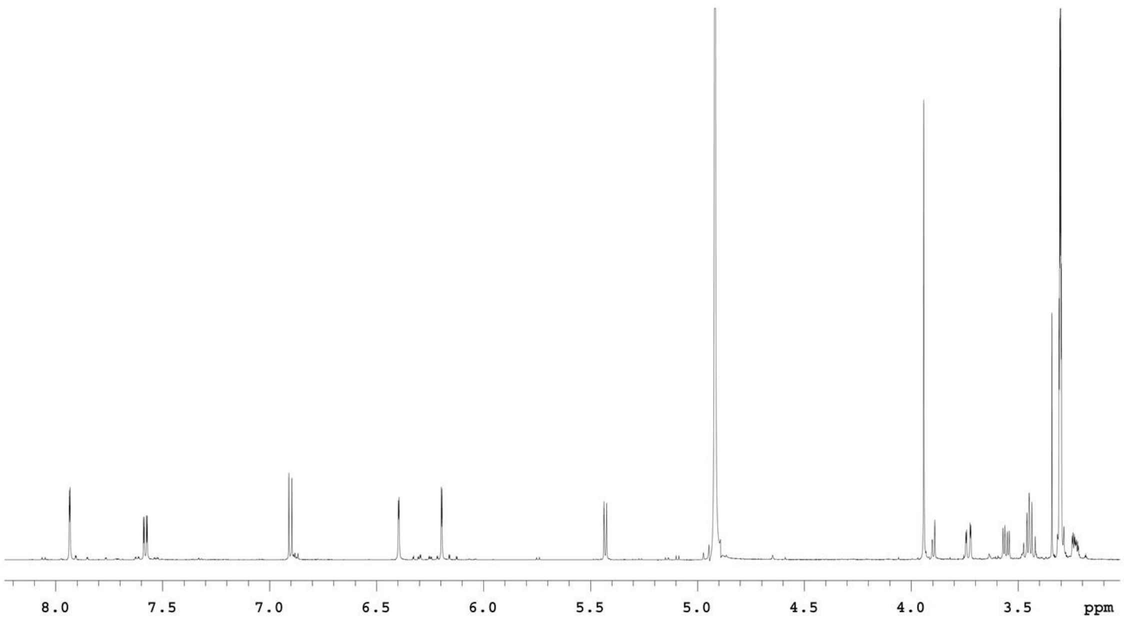

Figure S2. ^{1}H-NMR spectrum of compound **1** (isorhamnetin-3-*O*-glucoside)

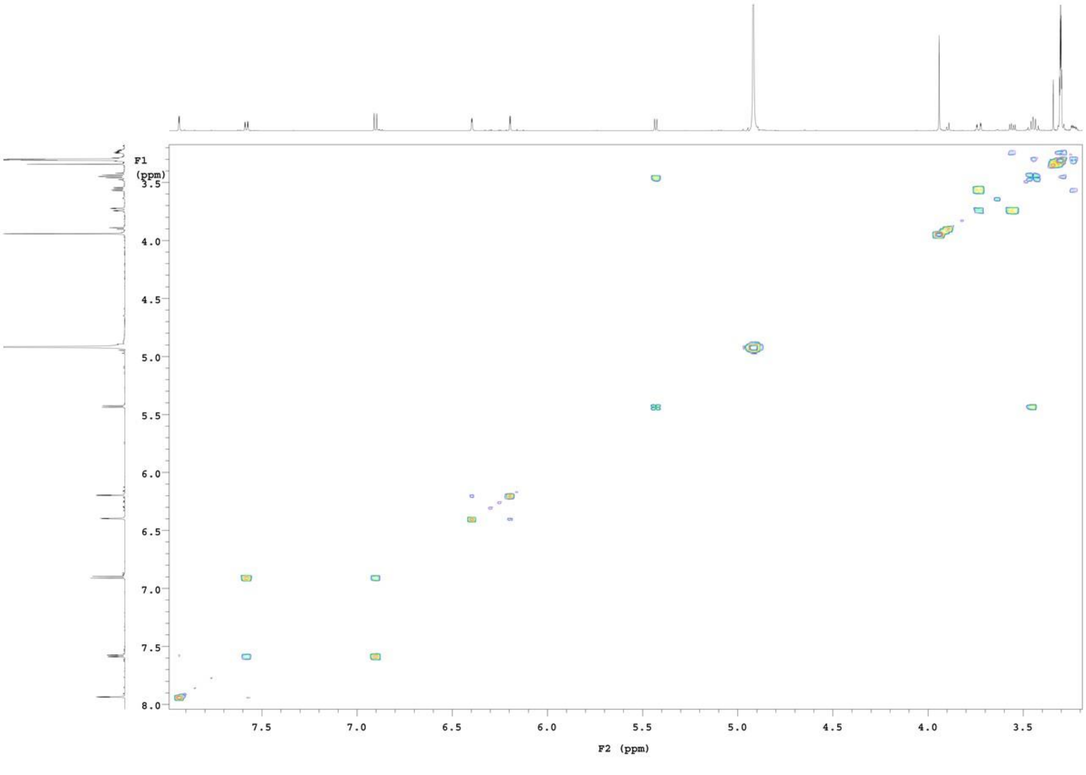

Figure S3. ^{1}H-^{1}H COSY spectrum of compound **1** (isorhamnetin-3-*O*-glucoside)

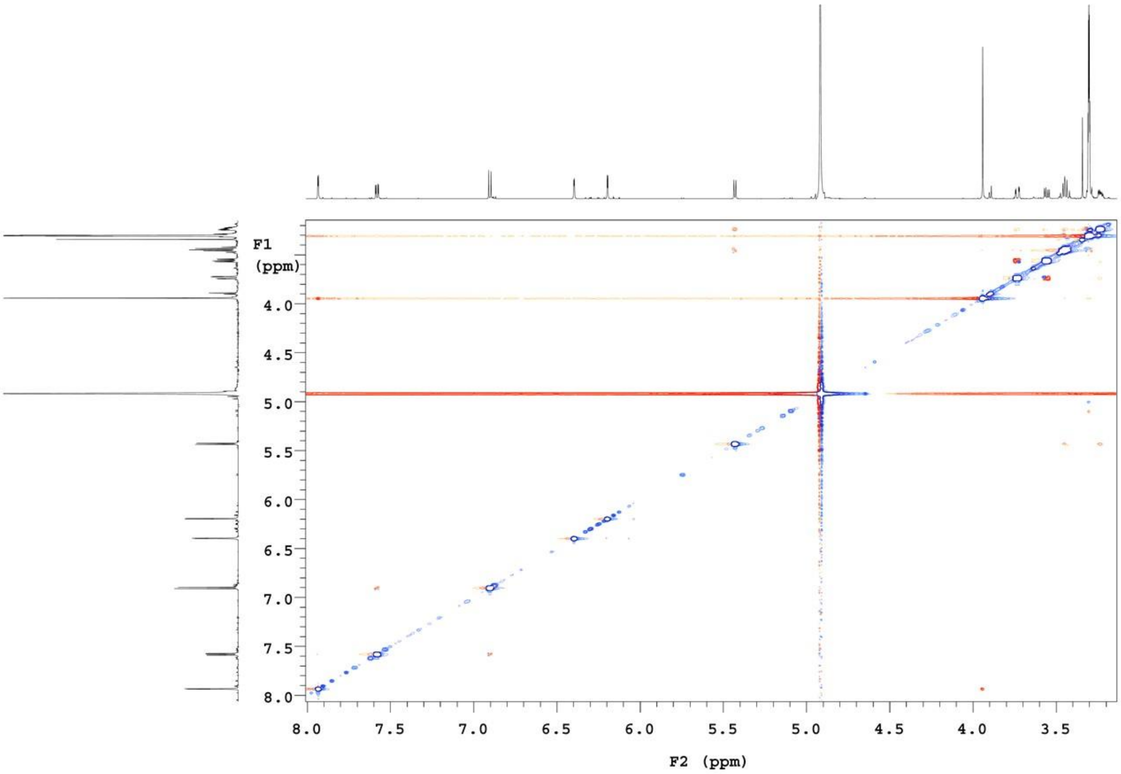

Figure S4. NOESY spectrum of compound **1** (isorhamnetin-3-*O*-glucoside)

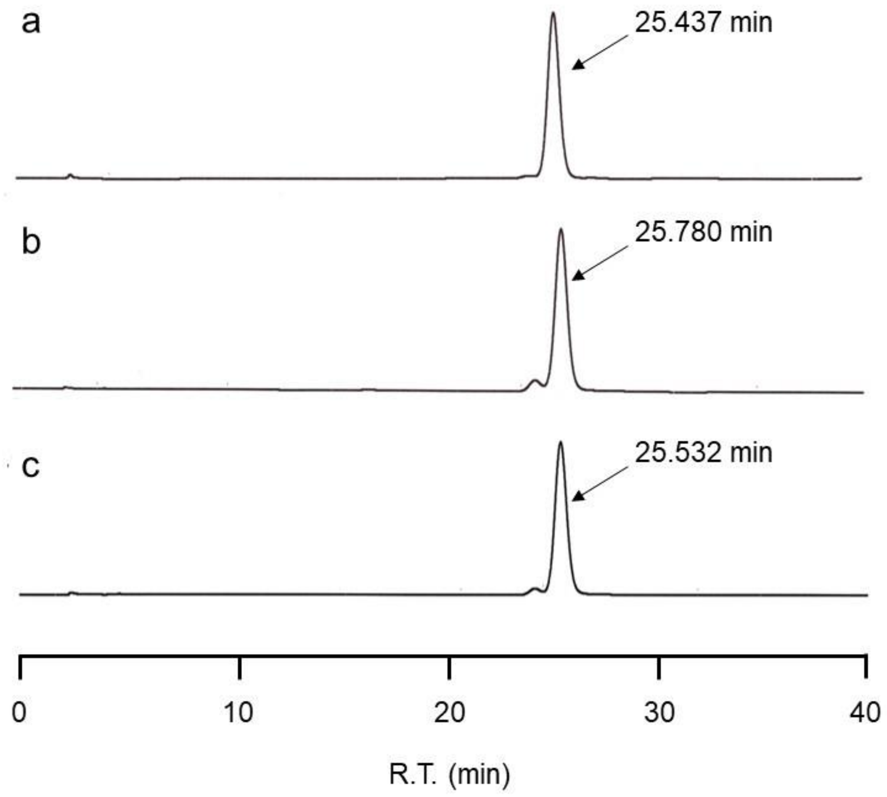

Figure S5. HPLC analyses of compound **1**. (a) HPLC analysis of compound **1**. (b) HPLC analysis of isorhamnetin-3-*O*-glucoside of standard. (c) HPLC co-chromatography analysis of compound **1** and isorhamnetin-3-*O*-glucoside of standard. HPLC analyses were performed on an Inertsil ODS-3 column (250 mm × 4.6 mm i.d., 5 μm, GL Sciences Inc., Tokyo, Japan) with MeOH/H_2O/acetic acid (40/59/1, *v/v/v*) at a flow rate of 0.7 mL/min. The absorbance at 254 nm was monitored for compound **1** and isorhamnetin-3-*O*-glucoside.

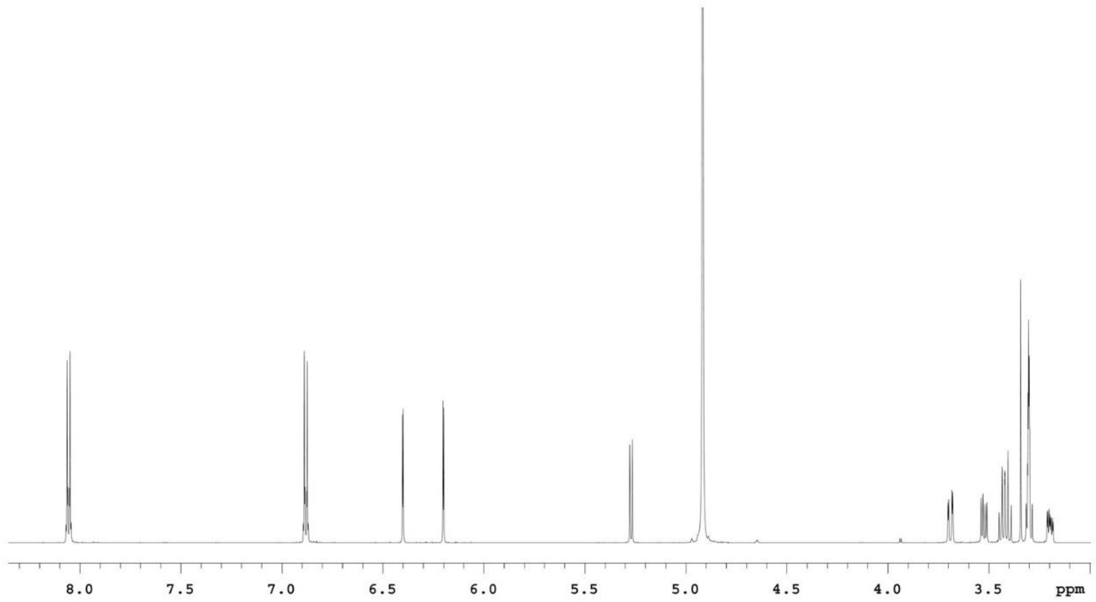

Figure S6. ^{1}H-NMR spectrum of compound **2** (astragalin)

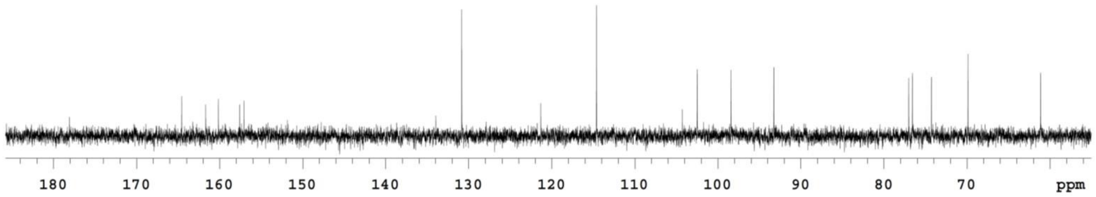

Figure S7. ^{13}C-NMR spectrum of compound **2** (astragalin)

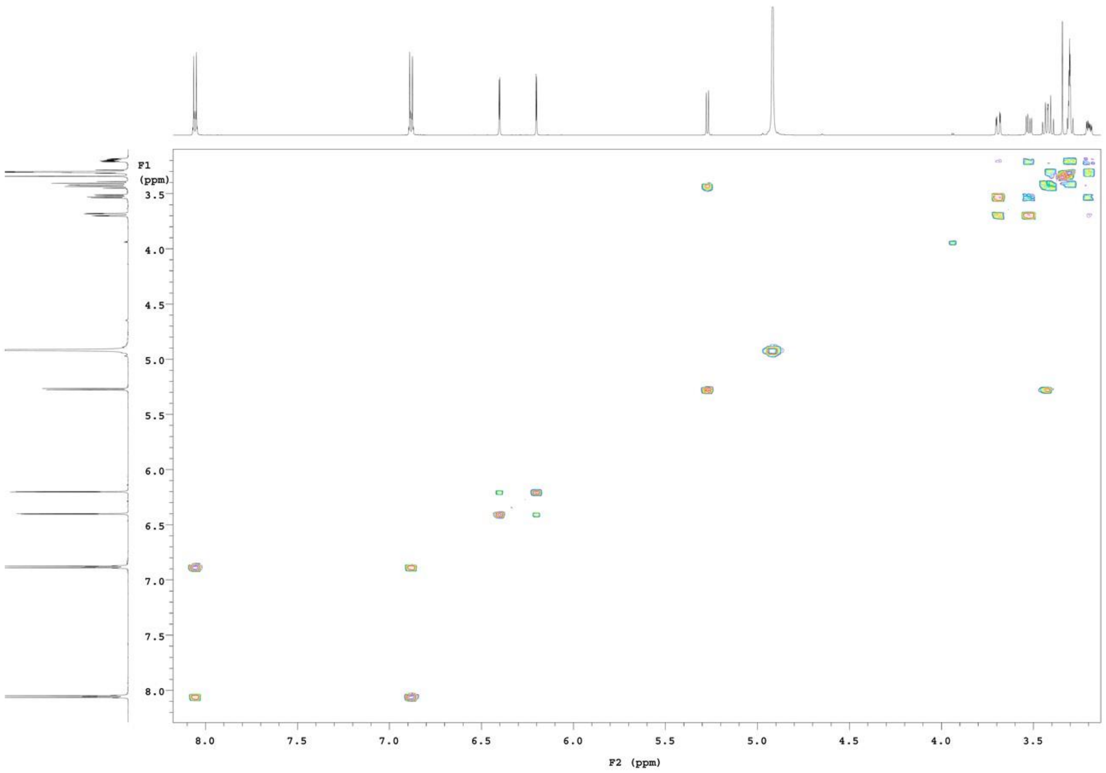

Figure S8. ^{1}H-^{1}H COSY spectrum of compound **2** (astragalin)

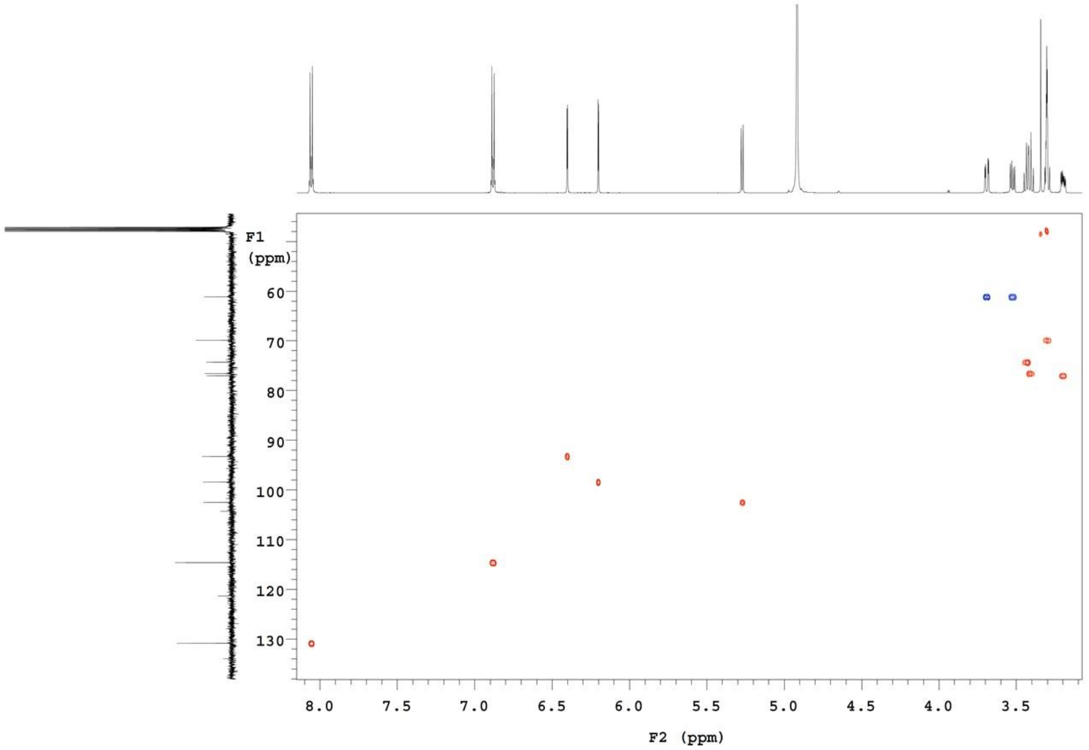

Figure S9. HSQC spectrum of compound **2** (astragalin)

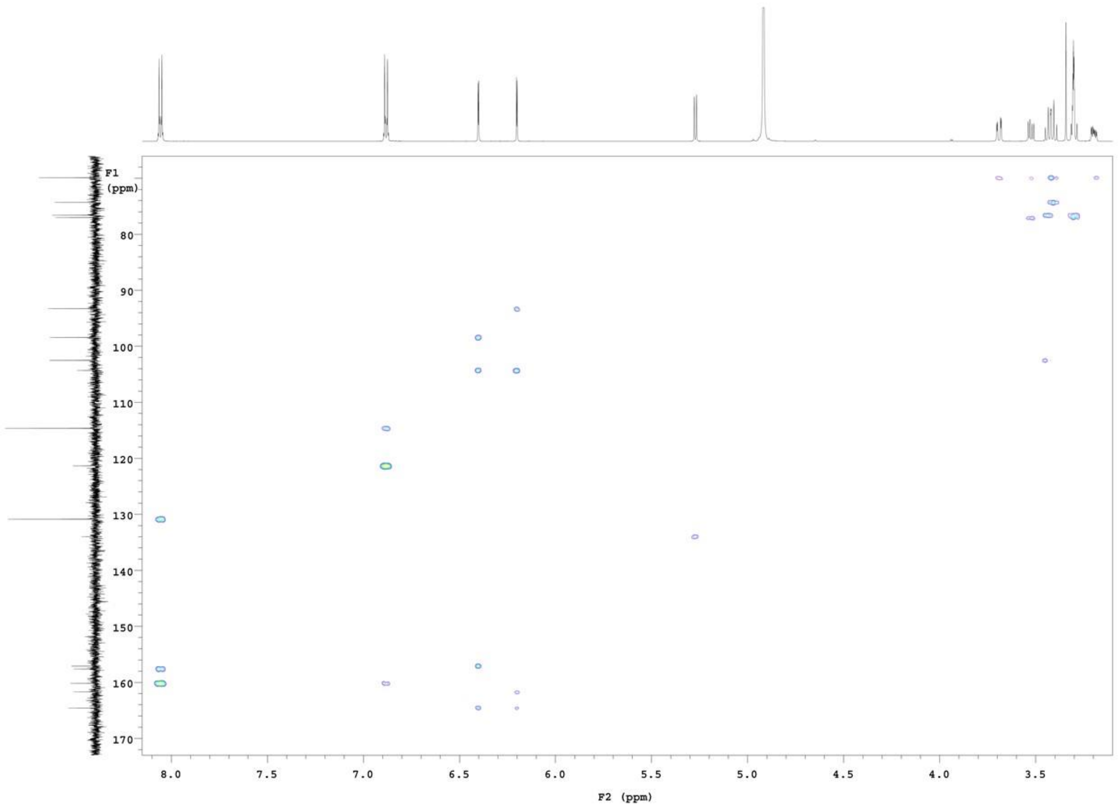

Figure S10. HMBC spectrum of compound **2** (astragalin)

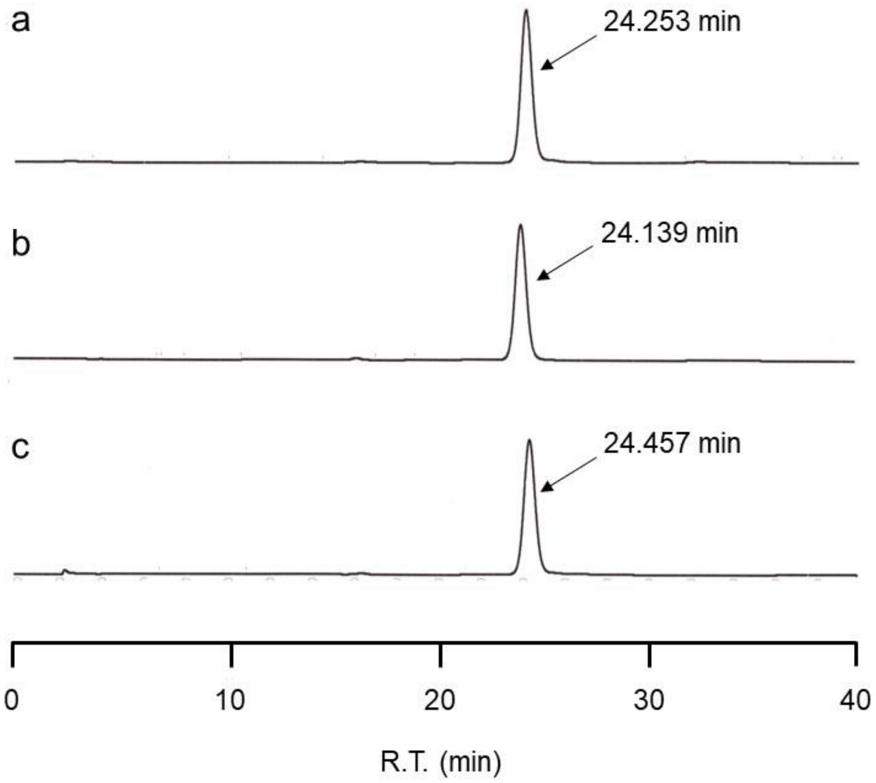

Figure S11. HPLC analyses of compound **2**. (a) HPLC analysis of compound **2**. (b) HPLC analysis of astragalin of standard. (c) HPLC co-chromatography analysis of compound **2** and astragalin of standard. HPLC analyses were performed on an Inertsil ODS-3 column (250 mm × 4.6 mm i.d., 5 μm, GL Sciences Inc., Tokyo, Japan) with MeOH/H_2O/acetic acid (40/59/1, *v/v/v*) at a flow rate of 0.7 mL/min. The absorbance at 254 nm was monitored for compound **2** and astragalin.
